# Purinergic Receptor Antagonists: A Complementary Treatment for Hypertension

**DOI:** 10.3390/ph17081060

**Published:** 2024-08-13

**Authors:** Rocio Bautista-Pérez, Martha Franco

**Affiliations:** 1 Department of Molecular Biology, Instituto Nacional de Cardiología Ignacio Chávez, México City 14080, Mexico; maria.bautista@cardiologia.org.mx; 2 Department of Cardio-Renal Pathophysiology, Instituto Nacional de Cardiología Ignacio Chávez, México City 14080, Mexico

**Keywords:** hypertension, angiotensin II, ATP, purinergic receptors

## Abstract

The treatment of hypertension has improved in the last century; attention has been directed to restoring several altered pathophysiological mechanisms. However, regardless of the current treatments, it is difficult to control blood pressure. Uncontrolled hypertension is responsible for several cardiovascular complications, such as chronic renal failure, which is frequently observed in hypertensive patients. Therefore, new approaches that may improve the control of arterial blood pressure should be considered to prevent serious cardiovascular disorders. The contribution of purinergic receptors has been acknowledged in the pathophysiology of hypertension; this review describes the participation of these receptors in the alteration of kidney function in hypertension. Elevated interstitial ATP concentrations are essential for the activation of renal purinergic receptors; this becomes a fundamental pathway that leads to the development and maintenance of hypertension. High ATP levels modify essential mechanisms implicated in the long-term control of blood pressure, such as pressure natriuresis, the autoregulation of the glomerular filtration rate and renal blood flow, and tubuloglomerular feedback responses. Any alteration in these mechanisms decreases sodium excretion. ATP stimulates the release of vasoactive substances, causes renal function to decline, and induces tubulointerstitial damage. At the same time, a deleterious interaction involving angiotensin II and purinergic receptors leads to the deterioration of renal function.

## 1. Introduction

During the last century, the treatment of hypertension has been focused on restoring several mechanisms involved in the development and persistence of hypertension. However, despite the advanced therapies presently offered for blood pressure control, their associated complications are far from being eliminated; consequently, cardiovascular disorders, including chronic renal disease, are often observed in patients with hypertension [1,2].

Most antihypertensive treatments have been mainly directed to block the renin–angiotensin system at its pathways, control hyperactivity of the sympathetic nervous system, restore cellular calcium alterations, and modify sodium reabsorption by the kidneys. In addition, a new treatment has been developed that blocks sodium–glucose transport with specific inhibitors. Such a variety of antihypertensive compounds should be enough to control blood pressure; however, factors associated with the control of hypertension are being ignored. Thus, insights into other pathophysiological mechanisms should be developed to preclude critical cardiovascular disorders [3].

Recent findings have improved knowledge of hypertension, which may result in a more rational treatment for this disease. The participation of the immune system, inflammation, and the development of oxidative stress are recognized as early responses to the elevation of blood pressure and the negative consequences of uncontrolled hypertension [4].

This review emphasizes the potential contributions of P2 purinergic receptors as well as the association between the tubulointerstitial inflammatory cells and ATP in the development of salt-sensitive hypertension as possible targets in the treatment of hypertension. Interested readers are directed to several recent, excellent, and comprehensive reviews covering the participation of the immune system, inflammation, and oxidative stress in hypertension [5,6,7].

The kidneys are fundamental to the regulation of blood pressure through intrinsic mechanisms such as pressure natriuresis, autoregulation of the glomerular filtration rate and plasma flow, tubuloglomerular feedback, and sodium excretion [8,9,10]; thus, they are essential organs to the initiation and continuance of hypertension. When renal function is normal, the mechanism of pressure natriuresis is intact. In this context, hypertension never occurs [10]. Conversely, a renal abnormality, such as a subtle renal lesion, may cause sustained hypertension [11]. The mechanisms involved in the development of renal injury in hypertension are currently better understood. Initially, it was thought that the systemic pressure was transmitted to the glomerulus and damaged it; this was due to an incapacity of the afferent arteriole to contract and regulate the pressure that reached the glomeruli [12]. Despite the development of hyperplasia and hypertrophy of the afferent arteriole, these are not sufficient to prevent microvascular damage in the capillary network, leading to the leakage of plasma and cells into the renal interstitium, which stimulates the development of tubulointerstitial injury [13,14]. However, inflammatory infiltration has been observed to occur since the initial stages of hypertension, and it is essential for the development of structural damage to the glomeruli, which results in end-stage renal disease [11]. Mild tubulointerstitial infiltration may induce renal dysfunction with sodium and water retention, leading to salt-sensitive hypertension [11,15]. In this regard, the experimental evidence suggests that the activation of purinergic receptors participates essentially in the pathophysiology of salt-sensitive hypertension through the constant release of vasoactive mediators [16], which promotes tubulointerstitial inflammation [11] and decreases pressure natriuresis [17].

## 2. Purinergic Receptors

ATP, the fundamental molecule providing an energy source within cells, possesses an extracellular membrane receptor system unrelated to energy production. In 1972, George Burnstock proposed ATP as a molecule whose extracellular effects fulfill the criteria to be mediated by membrane receptors [18]. Afterwards, it was confirmed that ATP membrane receptors exist [19,20]. Purinergic receptors are widely distributed in the human body and participate in the regulation of physiological and pathophysiological processes, since they are involved in inflammatory mechanisms as well as biochemical pathways that lead to apoptosis [20]. ATP breaks down into ADP, AMP, and adenosine. The first receptors described were P1 receptors, which are activated by adenosine; subsequently, P2 receptors activated by ATP were defined. There are two families of purinergic receptors, defined according to their pharmacological profiles: P2X receptors, which are ligand-gated membrane receptors designated 1 to 7, and P2Y receptors, a family of G-protein-coupled receptors that have seven transmembrane domains and are called 1 to 9, 12, 13, and 14 [21].

## 3. Purinergic Receptors in the Regulation of Blood Pressure

Purinergic signaling influences blood pressure at several levels and increases the sympathetic activity of peripheral nerves either directly or by acting on neurons in the brain stem. ATP is released by endothelial cells in response to shear stress and is involved in the release of nitric oxide. The activation of P2X3 receptors in the carotid body also influences sympathetic tone. In the kidneys, purinergic receptors possess tubular and vascular effects (Table 1) [22,23].

Kidney function involves intrinsic mechanisms such as the glomerular filtration rate, tubular transport, pressure natriuresis, and urinary sodium excretion, which are involved in the long-term control of blood pressure through adjustments to the extracellular volume. The extracellular fluid balance is fundamental for the regulation of blood pressure [8,9,10]. Among several renal mediators, ATP regulates the mechanisms mentioned above. P2Y2 receptor activation increases sodium excretion and decreases blood pressure by inhibiting the epithelial sodium channel (ENaC), thereby reducing sodium reabsorption and inducing an appropriate pressure-natriuresis response when the sodium concentration in the distal nephron is elevated [24,25]. ATP’s vasoactive effects through P2X receptors participate in adjustments to the renal vascular resistances, which are essential to maintaining glomerular capillary pressure and autoregulation. When ATP increases in the extracellular fluid, it is also augmented in the renal interstitial fluid and elevates the renal perfusion pressure [26,27,28]. In this regard, the stimulation of endothelial cells by shear stress releases ATP, and the continuous liberation of the nucleotide modifies the distribution of purinergic receptors, which are preferentially expressed in areas under hypoxic conditions [19,29,30].

**Table 1:** Distribution of purinergic receptors in the nephron.

Nephron Segments	P2X Purinergic Receptors	P2Y Purinergic Receptors
Proximal tubule	P2X1, P2X4, P2X5, P2X6	P2Y1, P2Y2, P2Y4, P2Y6
Loop of Henle, thick ascending limb	P2X4, P2X5	P2Y2, P2Y4
Loop of Henle, medullary thick ascending limb	P2X1, P2X4, P2X5, P2X6	P2Y1, P2Y2, P2Y4, P2Y6
Loop of Henle, thin descending limb	P2X4, P2X6	P2Y1, P2Y2
Loop of Henle, thin ascending limb	P2X4, P2X6	P2Y2, P2Y4
Distal tubule	P2X4, P2X5, P2X6	
Collecting duct	P2X1, P2X2, P2X3, P2X4, P2X5, P2X6	P2Y1, P2Y2, P2Y4, P2Y6
Afferent arteriole	P2X1, P2X7	P2Y1, P2Y2, P2Y6
Efferent arteriole		P2Y1
Glomeruli	P2X2, P2X4, P2X7	P2Y1, P2Y2, P2Y4, P2Y6
Perivascular capillaries and descending vasa recta	P2X7	P2Y1
Smooth muscle cells	P2X1, P2X2, P2X3, P2X7	P2Y1, P2Y2, P2Y4
Endothelium	P2X7, P2X4	P2Y1, P2Y2, P2Y6

Modified from Menzies et al. 2017 and Inscho et al. 2015 [21,26].

## 4. Purinergic Receptors in Hypertension

The activation of purinergic receptors may induce hypertension when extracellular ATP concentrations are elevated. Changes in the sympathetic tone and the stimulation of the renin–angiotensin system modify sodium excretion and may induce the contraction of pre- and post-glomerular arterioles. Furthermore, in Dahl salt-sensitive rats with stable hypertension [31] and angiotensin II-induced hypertensive rats, P2X1 receptors were overexpressed in the renal cortex, with no changes in P2Y1 receptor abundance [32]. P2X7 receptors have been observed in podocytes, endothelial cells, and mesangial cells in the glomeruli of hypertensive transgenic (mRen2) rats [33] and Dahl salt-sensitive hypertensive rats [31].

In angiotensin II-induced hypertensive rats, overexpression of the P2X1, P2X4, P2X7, and PY1 receptors has been observed in the intrarenal vessels, afferent arteriole, and macula densa [34,35]. It should be pointed out that purinergic receptors regulate several mechanisms engaged in the control of blood pressure, as mentioned above: pressure natriuresis [31], autoregulation of the glomerular filtration rate and blood flow [8,32,35], the tubuloglomerular feedback mechanism, and urinary sodium excretion [36,37,38]. In addition, purinergic receptors are important mediators of the progression of renal injury, at least in angiotensin II-mediated hypertension, since they stimulate vasoconstriction in the glomerular microcirculation. The afferent and efferent resistances are elevated, as well as the intraglomerular pressure, which results in a fall in the single-nephron and overall glomerular filtration rates [32,34].

Hypertensive kidneys are characterized by hypertrophy and hyperplasia of the renal vessels [39,40]. Since ATP is a powerful stimulus for the proliferation of smooth muscle cells in the renal vessels, these changes are mediated by the activation of purinergic P2X and angiotensin II AT1 receptors and are associated with interstitial injury: infiltration by lymphocytes and macrophages, the proliferation of mesangial cells and myofibroblasts around glomerular capillaries, and capillary rarefaction [41,42].

## 5. Beneficial Effects of Purinergic Receptor Blockade in Renal Microcirculation in Hypertension

**Figure 1:**
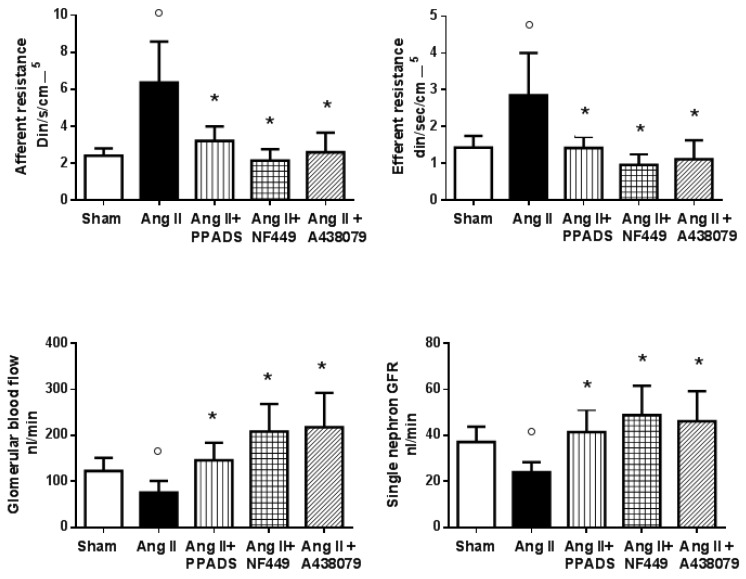
Renal hemodynamics in rats with Ang II-mediated hypertension (14 days, Ang II, 435 ng/kg/min) during an acute infusion of a broad purinergic receptor blocker (PPADS, 30 mg⋅kg^−1^) and specific P2X1 (NF 449, 30 nM⋅kg^−1^⋅h^−1^) and P2X7 (A 438079, 80 μm⋅kg^−1^) receptor antagonists. The purinergic antagonists induced a decrease in the afferent and efferent resistances (* < 0.05 to 0.019; ο < 0.05 vs. Sham) that produced a significant elevation in the glomerular blood flow; as a result of these changes, the single-nephron glomerular filtration rate returned to near-normal levels. These results show that, in Ang II-induced hypertension, the renal vasoconstriction induced by Ang II is associated with important actions of the P2X1 and P2X7 receptors (Modified by Franco et al. [32,34]).

In the context of P2X1 and P2X7 activation, vasoactive compounds such as inflammatory cytokines are released and are responsible for detrimental effects in renal microcirculation [43,44]. P2X7 receptor activation promotes a pro-inflammatory condition, since it induces the release of the cytokines Il-1β, Il-18, TNF-α, and MCP1, which are responsible for the stimulation of pathways that induce vasoconstriction [31,34,45,46]. Since P2X7 receptors are expressed in the smooth muscle cells of intrarenal vessels [36] and P2X1 receptors are expressed in the endothelial and smooth muscle cells, blockade with a purinergic antagonist was found to induce vasodilation in the renal microcirculation in hypertension [45,46]. When renal hemodynamics were evaluated after 14 days of infusion of angiotensin II in hypertensive rats, an acute blockade with broad (PPADS, pyridoxalphosphate-6-azophenyl-2′,4′-disulfonic acid) or specific P2X1 (NF 449, 4,4′,4,4-(carbonilbis(imino-5,1,3-benzenetriybis(carbonylimino)))tetrakis-benzene-l,3-disulfonic acid) and P2X7 (A438079, 6-difluoro-4-isopropyloxybenzyl alcohol) antagonists induced a vasodilatory response [32,34]. This was characterized by a fall in the resistances, an increase in the glomerular plasma flow, an increase in the ultrafiltration coefficient, and a return of the single-nephron glomerular filtration rate to nearly normal values (Figure 1) [34]. In the same model, when angiotensin II was administered at the same time as a P2X and P2Y purinergic antagonist (PPADS) for 14 days, the trophic effects of angiotensin II were prevented in the tubulointerstitium; indeed, inflammatory infiltration and afferent arteriole hypertrophy were significantly inhibited without changes in blood pressure neither of the angiotensin II concentrations [47]. 

## 6. Purinergic Receptors in Inflammation and Immunity

Inflammation is associated with ischemia and hypoxia, free radical oxygen species, and necrosis and apoptosis pathways [48,49,50]. Extracellular ATP is a potent stimulus for inflammation and may explain the immune activation that has been observed in hypertension. This issue is not currently understood. In this regard, to increase the ATP concentration in the interstitial space, the release of intracellular ATP is needed. This can be accomplished through cellular membrane channels called pannexins and connexins during the inflammatory process [51,52]. In the acute inflammatory reaction, ATP is metabolized to ADP and adenosine, then ectoenzymes such as ATPase, apyrase, alkaline phosphatase, and ectonucleotidases, decrease the concentration of extracellular ATP [50]. In contrast, in angiotensin II-induced hypertension, renal ecto-adenosine deaminase (which metabolizes adenosine) is decreased and induces the elevation of interstitial adenosine [53]. This fact should be pointed out, since the equilibrium between the vasodilator and vasoconstrictor effects of adenosine may be lost due to adenosine’s vasoactive properties [54]. It is important to mention that inflammatory cells possess P2X and P2Y receptors; this is the reason why interstitial ATP has chemoattractant effects [55]. In addition, inflammatory cells have the ability to enable the non-specific release of ATP when exposed to a deleterious stimulus, which induces the release of cytokines and chemoattractant factors [56,57,58]. The elevation of interstitial ATP modifies the expression and distribution of purinergic receptors when associated with the inflammatory reaction and induces the assembly of the NLRP3 (nucleotide-binding domain-like receptor pyrin domain containing 3) inflammasome [59,60,61], which is an essential step for the initiation of a proliferative reaction and the development of fibrosis in persistent hypertension [59].

The P2X7 receptor has been linked to the ensemble of the NRLP3 inflammasome, but the mechanism of the coupling is only partially known. In this regard, when ATP is elevated in the interstitial space, it induces protein phosphorylation, which, in turn, modulates protein ubiquitination and activates the NLRP3 inflammasome and the release of IL-1β and caspase-1 in macrophages and dendritic cells [60,61,62]; however, not all the effects of ATP are deleterious, since phagocytes and dendritic cells are also involved in tissue repair [63].

Regarding the inflammatory effect of ATP, it has been observed that immunosuppressor compounds such as mycophenolate mofetil (MMF), non-steroidal anti-inflammatory drugs (pentosan polysulfate), and genetic manipulations are associated with a reduction in tubulointerstitial infiltration and decreased renal injury [64,65,66]. The infiltration of macrophages is reduced along with NFkB, IL-1β, and TNFα [62], and treatment with the drugs mentioned above prevents the elevation of blood pressure [62]. The angiotensin II-induced hypertension model can be distinguished by producing severe hypertension associated with remarkable vasoconstriction; angiotensin II was administered for 14 days, followed by 5 weeks of a high-salt diet (NaCl: 4%) [67]. When angiotensin II and MMF were given simultaneously, barely borderline hypertension developed, a mild elevation of renal resistances was observed, and the glomerular blood flow and glomerular filtration rate were near normal levels; these changes were associated with a significant reduction in tubulointerstitial infiltration [64,65,67].

**Figure 2:**
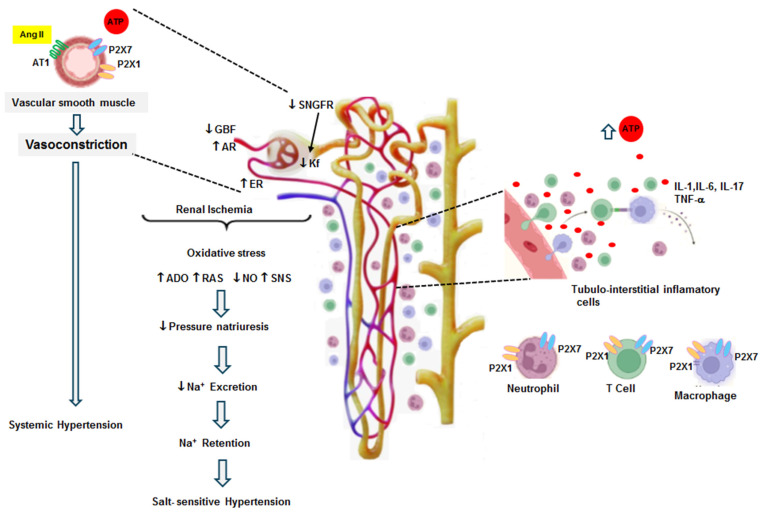
The vasoconstrictor effects of Ang II (14 days) and both P2X1 and P2X7 receptors are shown in this figure. Ang II infusion induced systemic hypertension and an elevation of interstitial fluid concentrations of ATP as well as local Ang II. The direct effect of Ang II associated with its regulatory response to hypertension produced renal vasoconstriction. The glomerular hemodynamics were characterized by an increase in the afferent resistance (AR) and efferent resistance (ER), which resulted in a decrease in the glomerular blood flow (GBF) and a diminished ultrafiltration coefficient (Kf). These alterations produced a reduction in the single-nephron glomerular filtration rate. These changes induced renal ischemia, leading to an overexpression of P2X receptors in the smooth muscle of intrarenal arterioles. Simultaneously, tubulointerstitial inflammatory cell infiltration contributed to a further elevation in interstitial ATP and the overexpression of P2X receptors in the intrarenal arterioles and on the surface of inflammatory cells. These changes induced the release of cytokines, growth factors, and chemoattractant factors; these factors exacerbated inflammatory cell infiltration and the intensity of renal vasoconstriction. Under these conditions, oxidative stress, the augmentation of adenosine (ADO), a decrease in nitric oxide (NO), an increase in the local production of Ang II (RAS), and the stimulation of sympathetic tone (SNS) developed. These alterations adjusted the sodium excretion and reduced pressure natriuresis, leading to decreased sodium excretion compared to the expected level for the elevation of blood pressure. This resulted in sodium retention, and salt-sensitive hypertension developed (modified from Graciano et al. 2008 [47] and Franco et al. 2019 [43]).

The development of hypertension and salt sensitivity and the activation of purinergic receptors can be summarized as follows: in situations of hyperactivity of the sympathetic nervous system, overstimulation of the renin–angiotensin system, or a genetic predisposition, some stress situations may induce a temporary elevation in blood pressure (11, 65, 66). When this elevation exceeds the limits of renal autoregulation (>140 mmHg), an increase in interstitial ATP occurs, as well as mild interstitial injury. The transmission of blood pressure damages the peritubular capillary walls, allowing the leakage of plasma and leukocytes to the tubulointerstitium; leukocytes induce local inflammation and enhance the severity of the microvascular and tubulointerstitial injury [65,67]. These alterations induce focal ischemia, cytokine release, upregulation of adhesion molecules, and capillary rarefaction, maintaining the inflammatory reaction [11]. In this context, the angiotensin II effects, the elevation of ATP, and tubulointerstitial inflammation become critical factors for the progression of renal injury [41,64,68,69,70]. These factors enhance the sensitivity of the renal mechanisms involved in the regulation of blood pressure and sodium excretion, leading to sodium retention (Figure 2) [71,72].

While the blood pressure increases, glomerular perfusion improves, since hypoxia and tubular ischemia decrease, resulting in the recovery of the oxygenation and perfusion of the kidneys [8,9]. At the same time, the elevation of glomerular blood flow stimulates nitric oxide release, leading to an increase in sodium excretion [8,9]. The blood pressure remains elevated as a result of the tubulointerstitial changes mentioned above, but the preservation of hypertension is required to maintain normal sodium excretion [45]. Then, salt sensitivity develops and sodium homeostasis is restored, but at the cost of hypertension [8,9]. Therefore, tubulointerstitial injury without glomerular damage is a condition frequently observed in the early stages of hypertension [67]. The vascular resistance increases initially in response to hypertension, inducing afferent arteriole hypertrophy. Despite these adaptative adjustments, after some time, hyperperfusion and glomerular hypertension develop, as well as glomerular capillary injury, resulting in a decrease in sodium excretion [73,74,75].

## 7. Perspectives

The treatment of hypertension with P2X antagonists has not yet been explored, but these antagonists are an attractive possibility for the control of blood pressure and the prevention of cardiovascular complications [76]. P2X7 antagonists are under development by several drug companies, and they have been tested in patients with inflammatory diseases, such as rheumatoid arthritis, neuroinflammation, pain, and cancer [77]. However, the implications of purinergic receptors in the pathophysiology of hypertension should be considered. Despite the favorable effects obtained in experimental hypertension models, the main limitations for the use of this treatment in patients will be identifying the correct moment to initiate the therapy during hypertension and selecting markers to evaluate the effects of the therapy, since inflammatory processes are chronic. Patients with uncontrolled hypertension or indicators of possible cardiovascular complications should benefit.

Alterations in purinoceptor function lead to various diseases [76], including neurological, rheumatic, cardiovascular, and renal diseases as well as cancer, among others [77]. To date, only P2Y12 antagonists have been used for their effect in antiplatelet therapy (suramin, pentoxifylline, clopidogrel, prasugrel, cangrelor, and ticagrelor). Newly developed compounds such as the P2Y2 agonist denufusol (cystic fibrosis) and the P2X7 antagonist AZD 9056 (Crohn’s disease) demonstrate the therapeutic potential of agonists and antagonists of purinergic receptors in various human diseases [78].

## 8. Conclusions

Uncontrolled hypertension is associated with renal vasoconstriction. It stimulates hypoxia, oxidative stress, autoimmunity, and inflammation, which are involved in the pathophysiological mechanisms that induce salt sensitivity. The particular combination of factors such as elevated sheer stress, a high interstitial ATP concentration, the activation of P2 receptors, and elevated renal interstitial Ang II collectively controls the release of interleukins and growth factors, which contributes to the development of hypertensive renal injury. In addition, the evidence presented in this review suggests that purinergic antagonists may help to prevent the progression of renal damage to chronic kidney disease in hypertensive patients.

## Data Availability

The original contributions presented in this study are included in the article; further inquiries can be directed to the corresponding author.
